# The association between HLA-B variants and amoxicillin-induced severe cutaneous adverse reactions in Chinese han population

**DOI:** 10.3389/fphar.2024.1400239

**Published:** 2024-05-28

**Authors:** Ting Wang, Jin Yang, Fanping Yang, Ye Cheng, Zichong Huang, Bei Li, Linlin Yang, Qinghe Xing, Xiaoqun Luo

**Affiliations:** ^1^ Children’s Hospital of Fudan University, Institutes of Biomedical Sciences of Fudan, Shanghai, China; ^2^ Department of Allergy and Immunology, Huashan Hospital, Fudan University, Shanghai, China; ^3^ Department of Dermatology, Huashan Hospital, Fudan University, Shanghai, China; ^4^ Research Center of Allergy and Diseases, Fudan University, Shanghai, China; ^5^ College of Chemistry, Green Catalysis Center, Zhengzhou University, Zhengzhou, China; ^6^ Department of Pharmacology, School of Basic Medical Sciences, Zhengzhou University, Zhengzhou, China

**Keywords:** amoxicillin, severe cutaneous adverse reactions, HLA-B, variants, whole-exome sequencing

## Abstract

**Background:**

Amoxicillin (AMX) is among the most prescribed and the best tolerated antimicrobials worldwide. However, it can occasionally trigger severe cutaneous adverse reactions (SCAR) with a significant morbidity and mortality. The genetic factors that may be relevant to AMX-induced SCAR (AMX-SCAR) remain unclear. Identification of the genetic risk factor may prevent patients from the risk of AMX exposure and resume therapy with other falsely implicated drugs.

**Methodology:**

Four patients with AMX-SCAR, 1,000 population control and 100 AMX-tolerant individuals were enrolled in this study. Both exome-wide and HLA-based association studies were conducted. Molecular docking analysis was employed to simulate the interactions between AMX and risk HLA proteins.

**Results:**

Compared with AMX-tolerant controls, a significant association of HLA-B*15:01 with AMX-SCAR was validated [odds ratio (OR) = 22.9, 95% confidence interval (CI): 1.68–1275.67; *p* = 7.34 × 10^−3^]. Moreover, 75% carriers of HLA-B*15:01 in four patients with AMX-SCAR, and the carrier frequency of 10.7% in 1,000 control individuals and 11.0% in 100 AMX-tolerant controls, respectively. Within HLA-B protein, the S140 present in all cases and demonstrated the strongest association with AMX-SCAR [OR = 53.5, *p* = 5.18 × 10^−4^]. Molecular docking results also confirmed the interaction between AMX and S140 of the HLA-B protein, thus eliminating the false-positive results during in association analysis.

**Conclusion:**

Our findings suggest that genetic susceptibility may be involved in the development of AMX-SCAR in Han Chinese. However, whether the HLA-B variants observed in this study can be used as an effective genetic marker of AMX-induced SCAR still needs to be further explored in larger cohort studies and other ethnic populations.

## Introduction

Amoxicillin (AMX) is among the most prescribed and the best tolerated antimicrobials worldwide (Sharland et al., 2018). However, AMX is also one of the most common drug allergy labels in the electronic health record, it can occasionally trigger severe cutaneous adverse reactions (SCAR), such as Stevens-Johnson syndrome (SJS), toxic epidermal necrolysis (TEN), drug reactions with eosinophilia and systemic symptoms (DRESS), and drug-induced acute erythroderma, with a significant morbidity and mortality (Aouam et al., 2010; Girelli et al., 2013; Pejcic et al., 2023). In time of the development of SCAR in combination medicines and antibiotic resistant settings, future exposure to all concurrently dosed drugs is contraindicated, seriously restricting the choice of candidate drugs for the patients (Sodhi et al., 2023). To date, SCAR is considered as immune-mediated delayed hypersensitivity reactions, due to drug-meditated immune response caused by cytotoxic T lymphocytes are specifically activated that occur until at least 3–4 days after of exposure to the causative drug (Chessman et al., 2008; Wei et al., 2012).

Advances in pharmacogenetics and pharmacogenomics have revealed the relationship between genetics and SCAR and excavated important molecules involved in the pathogenesis of drug hypersensitivity. A growing body of evidence suggested that HLA alleles are strongly associated with the drug-specific SCARs, and pretherapy screening for the risk HLA alleles could effectively reduce the occurrence of related SCARs. Notably, previous studies have identified the strong genetic associations between HLA-B*58:01 and allopurinol-induced SCAR(Hung et al., 2005) and HLA-B*15:02 and carbamazepine-induced SJS/TEN (Chung et al., 2004). Besides, HLA-B*57:01 (Mallal et al., 2008) and HLA-B*13:01 (Zhang et al., 2013) have also been found strong associations with abacavir hypersensitivity and dapsone hypersensitivity syndrome, respectively. The findings of these strong genetic associations have been applied to clinical screening to prevent the cases of severe drug hypersensitivity (Wang et al., 2021).

However, the genetic factors that may be relevant to AMX-induced SCAR (AMX-SCAR) remain unclear. The associations between genetic markers and beta-lactam antibiotics-induced adverse drug reactions have been reported. Yang et al. previously reported that *HLA-DRB* allele was found to be weakly associated with IgE-mediated-type hypersensitivity to penicillin in Chinese population (Yang et al., 2006). HLA-DRB1*10:01 allele was found to be associated with penicillin-induced immediate reactions (Nicoletti et al., 2021)and HLA-B*55:01 was related to penicillin-induced delayed reactions by genome-wide association study (Krebs et al., 2020) in European population. Romano et al. reported that HLA-DRB3*02:02 allele was strongly associated with penicillin-induced delayed hypersensitivity compared to immediate reaction in Italian population (Romano et al., 2022). Besides, Wattanachai et al. recently reported that six HLA alleles including HLA-A*01:01, HLA-B*50:01, HLA-C*06:02, HLA-DRB1*15:01, HLA-DQA1*03:01, and HLA-DQB1*03:02, were associated with the increased risk of beta-lactam antibiotics-related SCARs in Thai population (Wattanachai et al., 2023). To investigate the genetic predisposition to hypersensitivity reactions induced by AMX antibiotic to prevent patients from the risk of AMX exposure and resume therapy with other falsely implicated drugs in Chinese population, we carried out a case-control association study of patients with AMX-induced SCAR by using whole-exome sequencing (WES) and then further validated the results in AMX-tolerant population. Furthermore, molecular docking modeling was employed to elucidate the potential key amino acid residues of HLA molecules bound to AMX and the specific conformation pattern of ligand-receptor binding.

## Materials and methods

### Subject enrollment

Four cases with AMX-induced SCAR were recruited from Huashan Hospital of Fudan University in Shanghai, China from January 2006 to December 2022. Phenotypes of SCAR were classified according to the consensus definitions of RegiSCAR study criteria (Auquier-Dunant et al., 2002; Cacoub et al., 2011; Wang et al., 2018). The drug causality for each enrolled case was determined by the dermatologist using the algorithm of drug causality for epidermal necrolysis (ALDEN) score published by the RegiSCAR study group or the Naranjo algorithm (Naranjo et al., 1981; Sassolas et al., 2010; Kardaun et al., 2013). Data on patients’ demography, clinical patterns, causative drugs, treatments, and clinical outcomes were collected from patients’ case notes. Patients’ files with incomplete data were excluded. For general population and drug-tolerant controls, we collected clinical data and DNA samples of 1,000 individuals in general population without history of adverse drug reactions as the WES control group as well as 100 drug-tolerant patients who had received amoxicillin for several times and the cumulative duration was for more than 3 months without evidence of adverse reactions were enrolled as tolerant controls (Supplementary Table S1). The latency period between the intake of the drug and the onset of the skin eruption was recorded. Written informed consent has been collected from all subjects, and the study protocol was approved by the institutional review board of Department of Dermatology, Huashan Hospital.

### HLA genotyping

The whole-exome sequencing (WES) method include steps such as DNA sample extraction, library building, and sequencing, following the methods reported previously by (Jiang et al., 2022). Typing of HLA alleles were performed using HLA-HD version 1.3.0 (Kawaguchi et al., 2017) with sequence mapped to the reference sequence of six-digit HLA alleles from the IMGT/HLA database in default settings (Barker et al., 2023). High-resolution four-digit HLA-B allele genotyping was performed by Weihe Biotechnology (Jiangsu, China) using the polymerase chain reaction-sequence based typing (PCR-SBT) method.

### Molecular modeling

We conducted molecular docking studies via the Maestro (version 2019) software to investigate the interaction patterns between AMX and HLA protein. The protein and nucleotide sequences of HLA-B*15:01, HLA-B*46:01, HLA-B*15:02, and HLA-B*35:05 were obtained from the IMGT/HLA database. The structure of these four HLA protein molecules were obtained from SWISS-MODEL server, and HLA-B*15:01, HLA-B*15:02, HLA-B*35:05 and HLA-B*46:01 were modeled based on the following crystal structures, respectively: 5TXS (100% identity), 6UZM (100% identity), 7SIF (100% identity) and 4LCY (100% identity). AMX molecule was used as ligands and HLA molecule was used as receptors for docking modeling. The receptor molecule obtained the dominant conformation after repairing the missing residues and minimizing the energy using Maestro software. Ligprep module was used to generate 32 3D conformations of AMX molecules, and the optimal conformation was selected for modeling. The above receptor and ligand molecules were protonated at human physiological PH value. All parameters were set to the software default parameters. The docking simulation of the optimized ligand receptor was performed, and the docking models of the top 20 outputs were displayed. According to the docking scores and results, the optimal binding mode of AMX docking with each HLA molecule was selected.

### Statistical analysis

The statistical analyses were carried out using R version 4.2.3 (The R Foundation for Statistical Computing, Vienna, Austria). Individuals who tested positive for at least one copy of a specific allele were considered as carriers. Fisher exact test was applied to compare the carrier frequency between specific HLA allele for AMX-SCAR patients and population controls as well as AMX-tolerant controls. Overall odd ratios (ORs) with corresponding 95% confidence intervals (CIs) were calculated to quantify the association. When zero cell counts were included, ORs were calculated using Haldane’s modification, which adds 0.5 to all cells to accommodate possible zero counts to reduce bias in estimating ORs. In the HLA protein sequence analysis, the χ^2^ test and Fisher’s exact test were used to analyse the polymorphic position and amino acid residue between the AMX-SCAR group and two control groups, respectively. A two-tailed *p*-value of <0.05 was considered as statistically significant.

## Results

### Demographic and clinical characteristics of patients

Herein, we described four patients with AMX-SCAR recruited from the dermatology ward from Huashan Hospital of Fudan University between 2010 and 2021, who have been diagnosed with SJS, TEN, DRESS and Acute Erythroderma, respectively (Table 1). One out of four patients underwent oral AMX monotherapy, and the remaining three were on oral AMX combined with other drugs, for which we have ruled out other possible combined drugs as the culprit based on the medical history and ALDEN score or Naranjo algorithm. Latency period ranged from 1 to 9 days, with an average of 6.5 ± 2.6 days. Among them, three out of four patients had fever (≥37.5°C), 2 cases presented increased white blood cell count (≥0.5×10^9^/L), 1 case exhibited drug induced liver injury, and 2 cases had a history of penicillin allergy implying cross-reactivity with other penicillins.

**TABLE 1:** Clinical characteristics and HLA-B genotypes of patients with amoxicillin-induced severe cutaneous adverse reactions.

Gender/age	SCAR	Fever	WBC	EP	AST	ALT	ALP	GGT	Latency (days)	Allergic history	Combined drug	Hospitalization time (days)	*HLA-B*
M/60	DRESS	38°C	31.7↑	3.6↑	735↑	786↑	1,448↑	1,021↑	5	-	Niuhuang Jiedu Tablet	11	*13:01/* ***15:01***
F/28	TEN	39.5°C	13.0 ↑	0.5	17	35	27	11	9	-	Acetaminophen	14	***15:01*** */51:01*
F/49	Acute Erythroderma	-	9.9	0	14	22	46	13	1	Penicillin	-	6	***15:01*** */37:01*
F/56	SJS	37.5°C	12.1 ↑	0.8 ↑	11	12	28	7	4	Penicillin	chlorphenazine	9	*39:01/35:05*

Abbreviations: F, female; M, male; SCAR, severe cutaneous adverse reaction; DRESS, drug reaction with eosinophilia and systemic symptoms; SJS, Stevens-Johnson syndrome; TEN, toxic epidermal necrolysis; WBC, white cell count; EP, eosinophil; AST, aspartate transaminase; ALT, alanine transaminase; ALP, alkaline phosphatase; GGT, gamma-glutamyl transferase; HLA, human leukocyte antigen. The bold entries highlight that HLA-B*15:01 is positive in these patients.

### Associations of HLA alleles and amino acids with AMX-SCAR

The HLA analysis detected three carriers of HLA-B*15:01 in four patients with AMX-SCAR, and the carrier frequency of HLA-B*15:01 is 10.7% in 1,000 population controls and 11.0% in 100 AMX-tolerant controls, implying a significant association of HLA-B with AMX-SCAR (Supplementary Table S2, Table 2). Previous studies suggested that amino acid motifs rather than classical alleles were the major drives conferring disease risk (Illing et al., 2012; Raychaudhuri et al., 2012). In the present study, three out of four patients harbored HLA-B*15:01, suggesting it may play an important role in the pathogenesis of AMX-SCAR (Table 1). Then, we further analyzed the frequencies of amino acids in HLA-B proteins in cases and 100 AMX-tolerant controls, detecting 28 amino acids significantly enriched in patients (*p* < 0.05). Among them, S140 (*p* = 5.18 × 10^−4^) demonstrated the strongest association, followed by A48 (*p* = 4.45 × 10^−3^), L187 (*p* = 4.45 × 10^−3^), R121 (*p* = 6.84 × 10^−3^), W14 (*p* = 6.84 × 10^−3^), V17 (*p* = 6.84 × 10^−3^), and W180 (*p* = 7.34 × 10^−3^) (Supplementary Table S3).

**TABLE 2:** Frequencies of HLA alleles in patients with amoxicillin-induced severe cutaneous adverse reactions and amoxicillin-tolerant controls.

*HLA-B* genotype	Carrier, No. (%)		*P*	OR (95% CI)
	AMX-SCAR (4)	AMX-tolerant controls (100)		
B*13:01	1 (25)	10 (10)	0.3650	2.95 (0.05–41.08)
B*15:01	3 (75)	11 (11)	**7.34 × 10** ^**−3**^	22.9 (1.68–1275.67)
B*35:05	1 (25)	2 (2)	0.1120	14.99 (0.21–375.00)
B*37:01	1 (25)	4 (4)	0.1810	7.64 (0.12–125.78)
B*39:01	1 (25)	2 (2)	0.1120	14.99 (0.21–375.00)
B*51:01	1 (25)	11 (11)	0.3920	2.66 (0.05–36.68)

Abbreviations: HLA, human leukocyte antigen; AMX, amoxicillin; SCAR, severe cutaneous adverse reaction; OR, odds ratio; CI, confidence interval; Significant differences indicate that *p* < 0.05. The bold entries emphasize that HLA-B*15:01 indeed differ significantly between patients and amoxicillin-tolerant controls. Bold entries indicate that *p* < 0.05.

### In silico analysis and molecular docking

**FIGURE 1:**
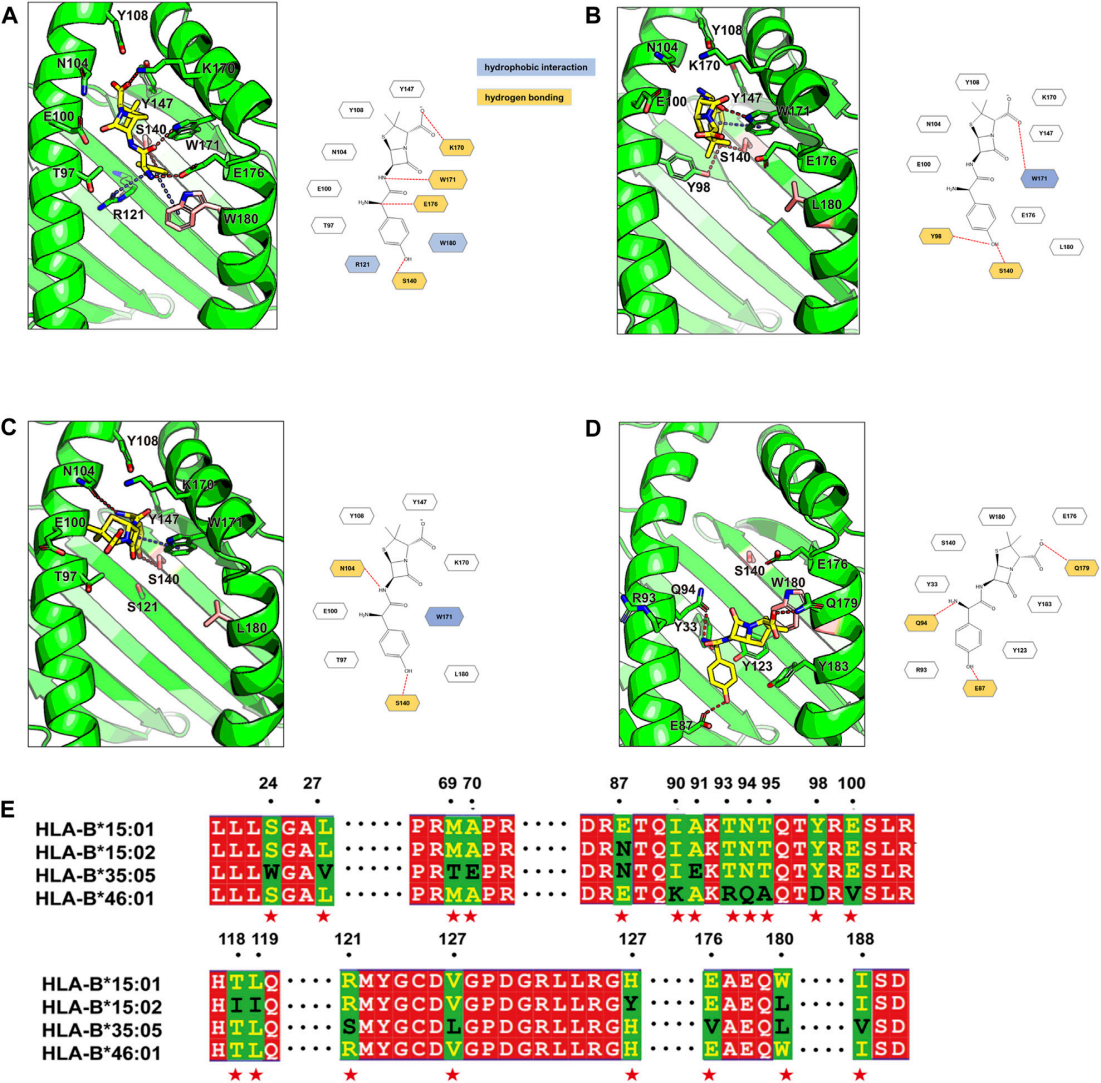
Binding modes of Amoxicillin in four HLA proteins and the sequence alignment. The binding modes of amoxicillin (AMX) in HLA-B*15:01 **(A)**, HLA-B*15:02 **(B)**, HLA-B*35:05 **(C)** and HLA -B*46:01 **(D)** are shown in the green cartoon representation. AMX (yellow carbons) and HLA residues (green carbons or pink carbons) that involve ligand bindings are shown in the stick representation. Hydrogen bonds are displayed as red dashed lines, π-π hydrophobic interactions and cation-π hydrophobic interactions are shown as blue dashed lines. For clarity, two-dimensional schematic representations of the AMX-HLA interactions are shown next to the three-dimensional models. In the sequence alignment **(E)** results, identical residues are shown with a red background; partially conserved residues are shown as white letter with a red background; Non-conserved residues are displayed with a green background, with the first type residue colored in yellow and the second type residue colored in black, and these residues are denoted as red stars at the bottom.

Given the strong associations of AMX-SCAR with specific amino acids of HLA-B, molecular modeling was conducted to further confirm the interaction between AMX and HLA-B proteins to eliminate false-positive results. The docking results revealed that amino acid at position 140 and 180 in HLA-B play a crucial role in binding to AMX. In HLA-B*15:01, S140, K170, W171, and E176 can form hydrogen bonds, R121 and W180 formed cation-π hydrophobic interactions with AMX. These combined effects gave rise to relatively low steric hindrance, which was conducive for AMX binding in the pocket with a low-energy conformation (Figure 1A). In HLA-B*15:02 (belongs to the same serotype and differs from HLA-B*15:01 by only 5 amino acids), W180 became L180, which greatly increased steric hindrance (Figure 1B). In HLA-B*35:05 (presents in patient 4), although the conversion of W180 to L180 slightly weakens the binding to the AMX molecule, S140 remains to be a key amino acid in forming hydrogen bonds. In addition, W171 and N104 also form hydrogen bonds, and this binding mode allows AMX to be surrounded by polar amino acids, promoting the formation of stable complexes in the dynamic binding process (Figure 1C). In HLA-B*46:01 (shows a high frequency in tolerant controls), AMX’s phenolic head is surrounded by polar amino acids such as E87, and the increased steric hindrance prevents AMX from remaining in a low-energy conformation in the pocket. Consequently, AMX loses the hydrophobic effect with W180 and the polar effect with S140 (Figure 1D). Overall, AMX tends to bind with S140 in the antigen-binding cleft of HLA-B, differing from unrelated HLA molecules HLA-B*46:01, which indicates that predisposition to AMX-SCAR is related to one essential amino acid residue in the peptide binding pocket of HLA-B.

### Genetic predictors of AMX-induced SCAR

For HLA-B proteins, amino acid residues S140, L187, A48, R121, W14, V17, I306, T329, and W180 are encoded by coding sequence variants C419, CT559-560, G142, G363, G41, G49, A916, A985, and T538, respectively. The distribution of these genetic variants in the AMX-SCAR and AMX-tolerant groups is consistent with amino acids. From the perspective of genetic variants as predictors of AMX-SCAR, where C419, CT559-560, G142, G363, G41, G49, A916, and A985 showed the highest sensitivity (up to 100%), followed by T538 with 75%. In terms of specificity, variant T538 exhibited the highest value (up to 89%), followed by C419 (87%), CT559-560 and G142 (76%), G363, G41, and G49 (73%), and A916 and A985 (72%). According to the current results, S140 of HLA-B or its genetic allele C419 (also known as rs4997052), present in all 4 subjects with AMX-SCAR but only 13 of the 100 AMX-tolerant controls, can serve as a clinical predictor of AMX-induced SCAR with a sensitivity of 100% and specificity of 87% (Table 3).

**TABLE 3:** Predictive efficiency of pharmacogenetic predictors for amoxicillin-induced severe cutaneous adverse reactions.

Predictor	Carrier, No. (%)		*P*	OR (95% CI)	Sensitivity (%)	Specificity (%)
	AMX-SCAR (4)	AMX-tolerant controls (100)				
HLA-B*15:01	3 (75)	11 (11)	7.34 × 10^−3^	22.9 (1.64–1262.54)	75	89
*T538*	3 (75)	11 (11)	7.34 × 10^−3^	22.9 (1.68–1275.67)	75	89
*C419*	4 (100)	13 (13)	5.18 × 10^−4^	53.5 (2.67–1072.29)	100	87

Abbreviations: AMX, amoxicillin; SCAR, severe cutaneous adverse reaction; OR, odds ratio; CI, confidence interval.

## Discussion

Although the incidence of SCAR is low, it may cause high mortality and permanent disability (Duong et al., 2017). Amoxicillin (AMX) is the most widely used penicillin all over the world, and is a World Health Organization (WHO)-designated core access antibiotic (Sharland et al., 2018). However, AMX is one of the leading causative drugs for life-threating SCAR. The results of the clinical characteristics of patients with AMX-SCAR showed that most of the patients were female, which was consistent with previous studies of the frequency of drug allergies (Blumenthal et al., 2019). Patients with the DRESS phenotype in this study had a latency period of 8 days, which is shorter than the typically reported for DRESS syndrome (3–6 weeks). Um et al. found that the latency period in antibiotic-induced DRESS was significantly shorter than in anticonvulsant-induced DRESS(Um et al., 2010). Drug-related skin changes generally begin to appear after 1–3 weeks of continuous drug use, and the latent period for cutaneous adverse drug reactions with SJS/TEN phenotype in this study was 8.5 days on average, which was consistent with recent reports (Lee et al., 2023; Pejcic et al., 2023).

Previous studies on the association between HLA and various diseases have suggested that amino acid variants, rather than classical alleles, are the main drivers of disease risk. Illing et al. reported that Asp114 and Ser116 in HLA-B*57:01 are key amino acids involved in the pathogenesis of abacavir hypersensitivity syndrome (AHS) (Illing et al., 2012). Raychaudhuri et al. revealed that five amino acids in three HLA proteins could explain most of the association between HLA and the risk of seropositive rheumatoid arthritis (Raychaudhuri et al., 2012). More recently, Chu et al. found that four amino acid positions could account for the associations of HLA with Graves’ disease (GD) in Han Chinese (Chu et al., 2018). In present study, key residue S140 in the antigen-binding pocket of HAL-B was the main factor interacting with AMX and had a notably higher risk of developing AMX-SCAR.

To date, genetic susceptibilities to AMX antibiotic-induced hypersensitivity remain unclear. There still lacks a strong and applicable genetic marker to prevent these adverse events. Through genetic association and molecular docking analysis, we identified one amino acid S140 of HLA-B or its corresponding genetic allele C419 as a potential predictor of AMX-SCAR. Prospective screening of the variant C419, coupled with an alternative drug treatment for carriers, may significantly decrease the incidence of AMX-SCAR in Han Chinese. The novelty of our work is also manifested in the first discovery of the causal and mechanistic linkage between S140 of HLA-B and AMX-SCAR, which can provide important clues for subsequent research. Nevertheless, the sample size of this study was limited due to challenges in recruiting SCAR cases with a clear cause, future studies with a larger sample size are required to confirm the fidelity of this genetic marker and explore the underlying mechanism.

## Data Availability

The data presented in the study are deposited in the NCBI Sequence Read Archive (SRA) repository: https://www.ncbi.nlm.nih.gov/sra/, accession number PRJNA1110861.
