# Supplementary material for: The association between HLA-B variants and amoxicillin-induced severe cutaneous adverse reactions in Chinese han population
Source: Front Pharmacol. 2024 May 28;15:1400239. doi: 10.3389/fphar.2024.1400239 (PMC11165025; doi:10.3389/fphar.2024.1400239)
Supplement: Supplementary file 1 [file Table1.DOCX]

Supplementary Material

# Supplementary Tables

**Supplementary Table 1.** Demographic and baseline clinical characteristics of amoxicillin-induced severe cutaneous adverse reaction patients and amoxicillin-tolerant controls from Han Chinese.

|  | Amoxicillin-induced SCAR  N=4 | Amoxicillin  tolerant control  N=100 | |
| --- | --- | --- | --- |
|  |  |  |  |
| Age, years, mean ± SD | 40.6±10.1 | | 45.7±11.2 |
| Sex, n (%) |  | |  |
| Male | 1 (25%) | | 39 (39%) |
| Female | 3 (75%) | | 61 (61%) |
| Deceased cases, No. (%) | 0 (0%) | | 0 (0%) |
| Underlying diseases, No. (%) | | |  |
| Tonsillitis | 1 (25%) | | 15 (15%) |
| Unknown fever | 2 (50%) | | 33 (33%) |
| Enteritis | 0 (0%) | | 1 (1%) |
| Acne | 0 (0%) | | 4 (4%) |
| Pneumonia | 0 (0%) | | 8 (8%) |
| Cellulitis | 1 (25%) | | 20 (20%) |
| Paronychia | 0 (0%) | | 1 (1%) |
| Lymphangitis | 0 (0%) | | 1 (1%) |
| Trichofolliculitis | 0 (0%) | | 10 (10%) |
| Neuritis | 0 (0%) | | 5 (5%) |
| Pleurisy | 0 (0%) | | 2 (5%) |

# Supplementary Table 2. Frequencies of HLA alleles with significant differences between patients with amoxicillin-induced severe cutaneous adverse reactions and population controls.

| **HLA genotype** | **Carrier, No. (%)** | | ***P*** | **OR (95% CI)** |
| --- | --- | --- | --- | --- |
|  | **AMX-SCAR**  **(n=4)** | **Population**  **controls (n=1000)** |  |  |
| *A*24:07* | 1 (25.0) | 5 (0.5) | 0.0237 | 63.54 (1.05-968.08) |
| *B*15:01* | 3 (75.0) | 107 (10.7) | 0.0047 | 25.04 (1.98-1304.39) |
| *B*35:05* | 1 (25.0) | 3 (0.3) | 0.0198 | 78.59 (1.27-1293.03) |
| *DQA1*05:06* | 1 (25.0) | 6 (0.6) | 0.0276 | 53.28 (0.90-785.75) |
| *DQB1*03:05* | 1 (25.0) | 2 (0.2) | 0.0119 | 151.78 (2.12-3991.70) |

Abbreviations: HLA, human leukocyte antigen; AMX, amoxicillin; SCAR, severe cutaneous adverse reaction; OR, odds ratio; CI, confidence interval; Significant differences indicate that *P* < 0.05.

**Supplementary Table 3.** Top 28 amino acids variants in the HLA-B protein associated with amoxicillin-induced severe cutaneous adverse reactions in the association analysis.

| **Multiallelic amino acids** | ***HLA-B* allele** | **Carrier, No. (%)** | | | ***P*** | **OR** |
| --- | --- | --- | --- | --- | --- | --- |
|  |  | **AMX-induced SCAR patients, (n = 4)** | **Tolerant controls, (n=100)** | |  |  |
| S140 | *15:01/35:05* | 4 (100) | | 13 (13) | 0.000518 | 53.5 |
| A48 | *15:01/35:05/51:01* | 4 (100) | | 24 (24) | 0.004453 | 25.3 |
| L187 | *15:01/35:05/51:01* | 4 (100) | | 24 (24) | 0.004453 | 25.3 |
| R121 | *13:01/15:01/37:01/39:01* | 4 (100) | | 27 (27) | 0.006843 | 21.6 |
| W14 | *13:01/35:05/37:01/51:01* | 4 (100) | | 27 (27) | 0.006843 | 21.6 |
| V17 | *13:01/35:05/37:01/51:01* | 4 (100) | | 27 (27) | 0.006843 | 21.6 |
| W180 | *15:01* | 3 (75) | | 11 (11) | 0.007342 | 22.9 |
| I306 | *15:01/35:05/37:01/51:01* | 4 (100) | | 28 (28) | 0.007821 | 20.6 |
| T329 | *15:01/35:05/37:01/51:01* | 4 (100) | | 28 (28) | 0.007821 | 20.6 |
| S349 | *15:01/35:05/37:01/39:01/51:01* | 4 (100) | | 30 (30) | 0.010086 | 18.7 |
| A35 | *13:01/15:01/35:05/51:01* | 4 (100) | | 34 (34) | 0.016053 | 15.5 |
| M36 | *13:01/15:01/35:05/51:01* | 4 (100) | | 34 (34) | 0.016053 | 15.5 |
| T69 | *35:05/37:01/51:01* | 3 (75) | | 17 (17) | 0.02188 | 14.1 |
| R2 | *13:01/15:01/35:05/37:01/51:01* | 4 (100) | | 38 (38) | 0.024343 | 13.1 |
| T4 | *13:01/15:01/35:05/37:01/51:01* | 4 (100) | | 38 (38) | 0.024343 | 13.1 |
| G15 | *13:01/15:01/35:05/37:01/51:01* | 4 (100) | | 38 (38) | 0.024343 | 13.1 |
| Y98 | *13:01/15:01/35:05/37:01/51:01* | 4 (100) | | 38 (38) | 0.024343 | 13.1 |
| V176 | *13:01/35:05/37:01/39:01* | 3 (75) | | 18 (18) | 0.025309 | 13.2 |
| F140 | *37:01/39:01* | 2 (50) | | 6 (6) | 0.028952 | 14.7 |
| T187 | *37:01/39:01* | 2 (50) | | 6 (6) | 0.028952 | 14.7 |
| T93 | *13:01/15:01/35:05/37:01/39:01/51:01* | 4 (100) | | 40 (40) | 0.029523 | 12.0 |
| N94 | *13:01/15:01/35:05/37:01/39:01/51:01* | 4 (100) | | 40 (40) | 0.029523 | 12.0 |
| T95 | *13:01/15:01/35:05/37:01/39:01/51:01* | 4 (100) | | 40 (40) | 0.029523 | 12.0 |
| S155 | *13:01/15:01/35:05/37:01/39:01/51:01* | 4 (100) | | 40 (40) | 0.029523 | 12.0 |
| E201 | *13:01/15:01/35:05/37:01/39:01/51:01* | 4 (100) | | 40 (40) | 0.029523 | 12.0 |
| T202 | *13:01/15:01/35:05/37:01/39:01/51:01* | 4 (100) | | 40 (40) | 0.029523 | 12.0 |
| M69 | *13:01/15:01* | 3 (75) | | 21 (21) | 0.037525 | 10.9 |
| A70 | *13:01/15:01* | 3 (75) | | 21 (21) | 0.037525 | 10.9 |

Abbreviations: HLA, human leukocyte antigen; AMX, amoxicillin; SCAR, severe cutaneous adverse reaction; OR, odds ratio; Significant differences indicate that *P* < 0.05.
